# Isolation and Characterization of Renal Erythropoietin-Producing Cells from Genetically Produced Anemia Mice

**DOI:** 10.1371/journal.pone.0025839

**Published:** 2011-10-11

**Authors:** Xiaoqing Pan, Norio Suzuki, Ikuo Hirano, Shun Yamazaki, Naoko Minegishi, Masayuki Yamamoto

**Affiliations:** 1 Department of Medical Biochemistry, Tohoku University Graduate School of Medicine, Sendai, Miyagi, Japan; 2 School of Nursing, Miyagi University, Sendai, Miyagi, Japan; The National Institute of Diabetes and Digestive and Kidney Diseases, United States of America

## Abstract

Understanding the nature of renal erythropoietin-producing cells (REPs) remains a central challenge for elucidating the mechanisms involved in hypoxia and/or anemia-induced erythropoietin (Epo) production in adult mammals. Previous studies have shown that REPs are renal peritubular cells, but further details are lacking. Here, we describe an approach to isolate and characterize REPs. We bred mice bearing an *Epo* gene allele to which green fluorescent protein (GFP) reporter cDNA was knocked-in (*Epo^GFP^*) with mice bearing an *Epo* gene allele lacking the 3′ enhancer (*Epo^Δ3′E^*). Mice harboring the mutant *Epo^GFP/Δ3′E^* gene exhibited anemia (average Hematocrit 18% at 4 to 6 days after birth), and this perinatal anemia enabled us to identify and purify REPs based on GFP expression from the kidney. Light and confocal microscopy revealed that GFP immunostaining was confined to fibroblastic cells that reside in the peritubular interstitial space, confirming our previous observation in *Epo-GFP* transgenic reporter assays. Flow cytometry analyses revealed that the GFP fraction constitutes approximately 0.2% of the whole kidney cells and 63% of GFP-positive cells co-express CD73 (a marker for cortical fibroblasts and *Epo*-expressing cells in the kidney). Quantitative RT-PCR analyses confirmed that *Epo* expression was increased by approximately 100-fold in the purified population of REPs compared with that of the unsorted cells or CD73-positive fraction. Gene expression analyses showed enrichment of *Hif2α* and *Hif3α* mRNA in the purified population of REPs. The genetic approach described here provides a means to isolate a pure population of REPs, allowing the analysis of gene expression of a defined population of cells essential for Epo production in the kidney. This has provided evidence that positive regulation by HIF2α and negative regulation by HIF3α might be necessary for correct renal *Epo* induction. (282 words)

## Introduction

Erythropoietin (Epo) governs mammalian erythropoiesis. Epo is a glycoprotein hormone mainly produced in the kidney and liver in response to changes in tissue oxygen tension. Epo regulates erythropoiesis by supporting the survival of erythroid progenitors and stimulating their differentiation and proliferation in bone marrow, hence increasing the oxygen-carrying capacity of blood [1]. Lack of Epo during mouse development leads to lethality at embryonic day 13.5 (E13.5) due to severe anemia [2] and over- or under-production of Epo results in polycythemia or anemia clinically [1]. Epo production is considered to be controlled primarily at the level of gene transcription and *Epo* gene expression is strictly regulated in a tissue/cell-specific and hypoxia/anemia-induced manner [3]–[7].

Several tissues have been reported to express the *Epo* gene; but the ability to produce substantial amounts of Epo during hypoxia/anemia is restricted to the fetal liver and adult kidney [4]–[8]. The kidney plays a major role in oxygen sensing and contributes ∼90% of plasma Epo in adult animals [9]. However, difficulties in identification and purification of the renal Epo-producing cells (REPs) have limited the understanding of the mechanism for controlling Epo production in kidney. REPs are frequently reported to be peritubular fibroblast-like cells in kidney [6], [10], [11]; and a hypoxia-dependent Epo-producing cell line derived from human renal cancer was also described recently to exhibit fibroblast-like phenotype [12]. However, further details remain to be elucidated [5], [7], [13].

Current knowledge of the molecular mechanisms of oxygen-sensing and renal *Epo* gene expression has been extrapolated mostly from in vitro studies in hepatoma cell lines [14]–[16]. These studies have suggested that hypoxia responsiveness of the *Epo* gene depends on an enhancer containing hypoxia-responsive elements (HREs) located in the 3′ flanking region of the gene (3′ enhancer), to which the hypoxia-inducible transcription factor (HIF) 1 binds. HIF1 is composed of two subunits, HIF1α and HIF1β. HIF1β is constitutively expressed, but HIF1α expression, almost absent in normoxia, is increased during hypoxia. Under normoxic conditions, HIF1α is hydroxylated at two proline residues by specific prolyl-4-hydroxylases (PHD1–3) that allow the E3 ubiquitin ligase von Hippel-Lindau (pVHL) to bind to HIF1α and mark it for proteasomal degradation. In addition, HIF1α is regulated by the aspargine hydroxylase factor inhibiting HIF1 (FIH1), which inhibits p300/CBP (CREB-Binding Protein) binding to HIF1α. The activities of PHD and FIH1 are basically dependent on cellular oxygen concentration and thus qualify as cellular oxygen sensors. Low oxygen tension causes inactivation of PHDs and FIH1, allows HIFα to accumulate, forms active transcription factor-complex HIF with HIF1β, recruits transcriptional cofactors, and initiates the transcription of hypoxia responsive genes including the *Epo* gene. Thus the PHD/pVHL/HIF system likes to be the oxygen-sensing pathway regulating *Epo* gene transcription [17].

However, recent clinical and *in vivo* studies have suggested a new layer of complexity to the mechanisms involved in the cellular response to hypoxia/anemia. Evidence from mouse models and hereditary erythrocytosis in humans has revealed that HIF2α rather than HIF1α plays a vital role in oxygen-regulated erythropoiesis and renal Epo production is probably regulated by PHD2/pVHL/HIF2α pathway [13], [18], [19]. There are three HIFα family members: HIF1α, HIF2α, and HIF3α, which share a number of similarities *e.g.* DNA-binding sequence, oxygen-dependent hydroxylation. Unlike ubiquitously expressed HIF1α, expression of HIF2α and HIF3α is limited to several tissues [20]. Both HIF1α and HIF2α activate transcription, while HIF3α negatively regulates HIF1α and HIF2α activity [20]–[22]. There is no literature on HIF3α's role in hematopoiesis thus far.

In order to clarify the whole picture of *Epo* gene regulation, we have generated a panel of mouse lines. First, we genetically deleted the 3′ enhancer (referred to as the *Epo^Δ3′E^ allele*) and showed that this enhancer is necessary for hepatic *Epo* expression during the perinatal period {E17–postnatal day 13 (P13)} but dispensable for renal *Epo* expression after birth. Mice homozygous for the targeted allele (*Epo^Δ3′E/Δ3′E^*) are viable and fertile, but exhibit anemia during late-embryonic and newborn stages [23]. Then, using a 180-kb *Epo* transgene with a green fluorescent protein (GFP) reporter (*Epo-GFP*), we recapitulated tissue-specific, hypoxia-inducible GFP expression in kidney and liver tissue of mouse. Mutation studies on the transgene indicated that GATA factors are required for suppression of ectopic expression of the gene, but not essential for the *Epo* gene induction in REPs [6]. Also, we developed GFP knock-in mice (*Epo^GFP/wt^*) by homologous recombination in mouse embryonic stem cells (NS and MY, unpublished data). By examining these mouse lines, we identified GFP-labeled REPs as a population of peritubular interstitial cells in the kidney after birth.

Taken together, all these data *in vivo* strongly imply novel mechanism(s) and necessitate detailed studies on REPs to explore a specific oxygen-sensing pathway underlying the hypoxia-induced Epo production in the kidney.

Fluorescence activated cell sorting (FACS) of GFP expressing cells has been widely used for the isolation of hematopoietic stem cells in our laboratory [24], [25]. To make the link between the molecular and cellular mechanisms of hypoxia-induced *Epo* expression, in this paper, we have addressed controversial issues in REPs using cell-sorting techniques. Our *Epo-GFP* transgenic mice are a source of REPs isolation, but a pretreatment to induce anemia is not always successful for stable GFP expression in the kidney [6]. We therefore generated *Epo^GFP/Δ3′E^* mice, in which REPs were labeled with GFP as a result of neonatal anemia caused by genetic modifications. Taking advantage of the strong GFP expression in anemic newborns carrying the 3′ enhancer deletion, we have purified by FACS a cell population responsible for anemia/hypoxia induced *Epo* expression in the kidney.

## Results

### Generation of *Epo^GFP/Δ3′E^* Mice

**Figure 1:**
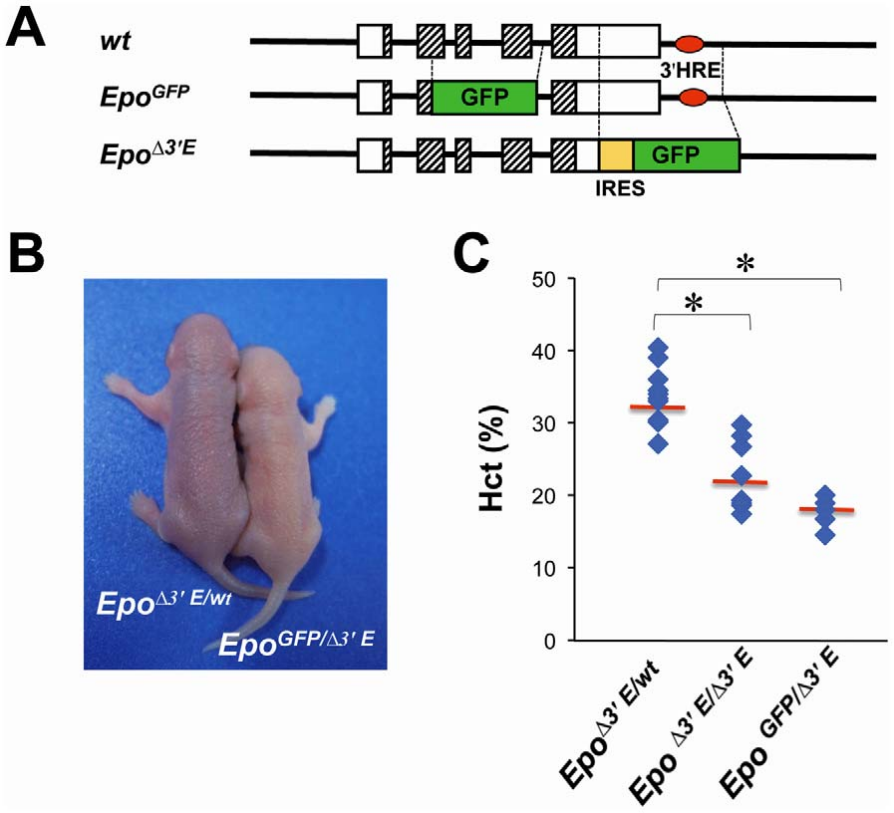
Generation of *Epo^GFP/Δ3′E^* mouse. **A** Diagram shows structures of the wild type (*wt*, upper), *Epo^GFP^* (middle) and *Epo^Δ3′E^* (lower), respectively. IRES, internal ribosomal entry site. **B**,**C** An *Epo^GFP/wt^* heterozygote and an *Epo^Δ3′E/Δ3′E^* homozygote were bred to obtain the compound *Epo^GFP/Δ3′E^* genotype. *Epo^GFP/Δ3′E^* mice exhibited transient anemia during the perinatal stage: **B** at P5 looked pale; **C** presented a decreased Hct value even lower than that of *Epo^Δ3′E/Δ3′E^* at P4–P6 stage. The *Epo^Δ3′E/wt^* littermates were used as a normal control. *p<0.05.

We first tried to define REPs by GFP fluorescence, which truthfully reflects endogenous *Epo* expression. We have established *Epo^GFP/wt^* mice by homologous recombination. In our *Epo^GFP/wt^* mice, the *Epo* locus was targeted by replacing the 3′ part of exon 2 through exon 4 with GFP (Figure 1A, middle panel). The mice homozygous for *Epo^GFP/GFP^*, which lacked a functional *Epo* gene, died around E13.5 from anemia, corresponding with the previous report on a *Epo* gene knockout mouse [2]. The heterozygous animals (*Epo^GFP/wt^*) were healthy and fertile; and distinct GFP expression, mimicking the endogenous *Epo* expression pattern, was observed in the kidney and liver under hypoxia/anemia conditions (NS and MY, unpublished data).

For steady *Epo* induction, we set out to genetically produce an anemia model by taking advantage of our established *Epo^Δ3′E/Δ3′E^* mice (Figure 1A, lower panel). As we previously reported in *Epo^Δ3′E/Δ3′E^* mice, deletion of the 3′ enhancer provokes transient anemia at late embryonic and neonatal stages due to defect in hepatic Epo production and erythropoiesis. This anemic phenotype is recovered in 2 weeks after birth when major Epo production site switches from the liver to kidney [23]. Crossing an *Epo^GFP/wt^* heterozygote with a mouse homozygous for *Epo^Δ3′E/Δ3′E^* allele, we generated *Epo^GFP/Δ3′E^* compound offspring. The compound mice basically showed a similar phenotype to that of their *Epo^Δ3′E/Δ3′E^* parents: anemia persisting after birth and recovered in the juvenile stage. *Epo^GFP/Δ3′E^* newborns were severely pale compared with *Epo^Δ3′E/wt^* littermates (Figure 1B). At P4–6, the Hematocrit (Hct) of the *Epo^Δ3′E/Δ3′E^* and *Epo^GFP/Δ3′E^* newborns were 22.7±5.3 and 18.0±2.0 (%), respectively (Figure 1C). At the same stage, the Hct of the *Epo^Δ3′E/wt^* control was 32.0±4.1%; this value is comparable with that of wild type (data not shown). Increased *Epo* mRNA could be detected in the kidney of the *Epo^GFP/Δ3′E^* newborns by quantitative (q) RT-PCR (see below).

### Cellular distribution of GFP expression in anemic neonatal kidneys

**Figure 2:**
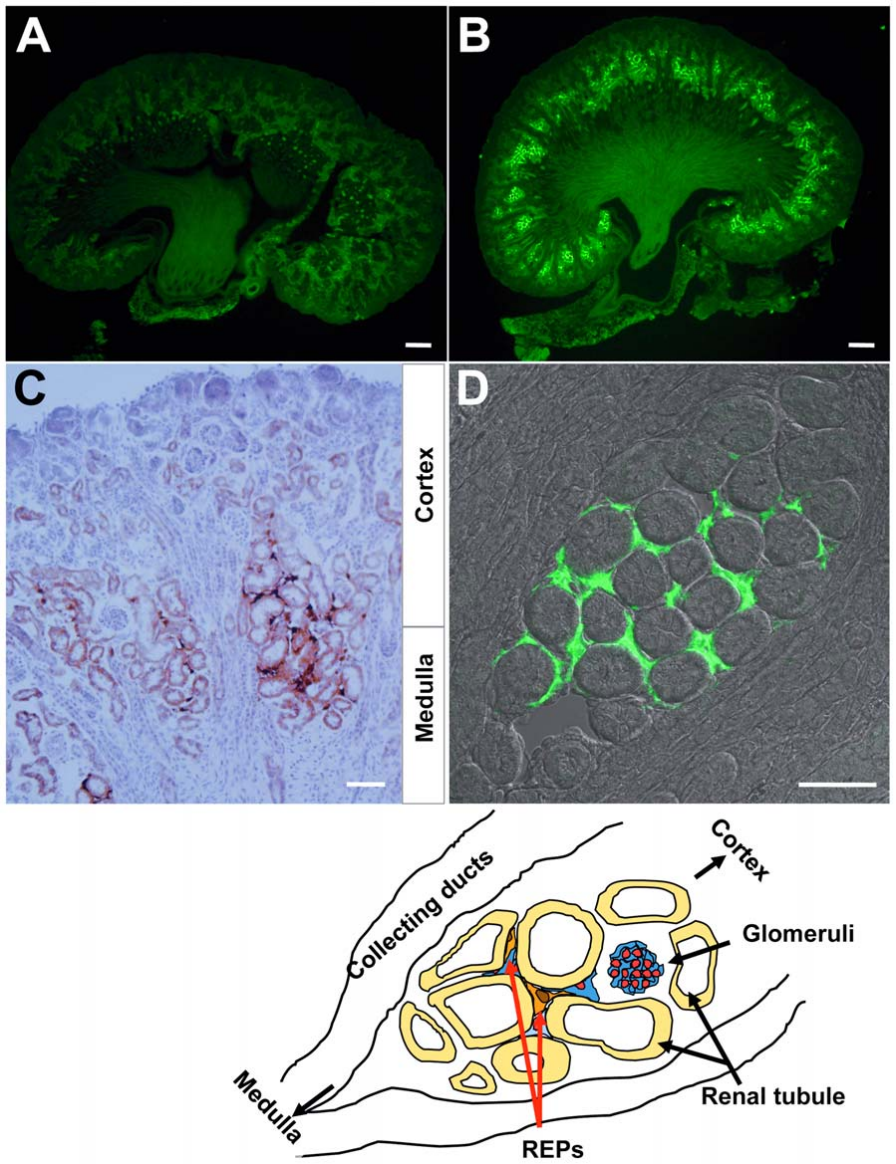
GFP expression in the anemic kidney of *Epo^GFP/Δ3′E^* mouse. Representative photos of P5 neonates: kidney slices **A** from a nonanemic *Epo^Δ3′E/wt^* littermate as a negative control; **B**–**D** from *Epo^GFP/Δ3′E^* newborns. Light and confocal microscopy revealing the GFP signal concentrated in the deep-cortex∼outer-medulla region (**B**), where the Epo-producing peritubular interstitial cells are localized (**C**,**D**). Anti-GFP signals: green (Alexa 488), and dark-brown (diaminobenzidine). Scale bars: 25 µm. The sketch at the bottom illustrates peritubular localization of the GFP-expressing cells in the kidney.

We then examined newborn (P4–6) kidneys to see GFP expression by immunostaining studies. In microscopic observation of the kidney section with anti-GFP immunostaining, fluorescence was minimal in control kidney samples from P4–6 *Epo^Δ3′E/wt^* littermates, but was prominent in the kidneys from the *Epo^GFP/Δ3′E^* mice (Figure 2A,B). GFP fluorescence was also directly detected in fixed *Epo^GFP/Δ3′E^* kidney slices by confocal microscopy (data not shown). GFP signals were confined to the cortex-medulla junction and focally distributed along the curve of the kidney at P4–6 (Figure 2B). GFP-expressing cells were stellate-shaped, nesting around proximal tubules in the deep cortical labyrinth and outer strip area of the kidney (Figure 2C,D). These findings are consistent with previous descriptions [5], [6], [26], [27] and indicate that the GFP expression reflects the endogenous expression of the *Epo* gene in kidneys of anemic *Epo^GFP/Δ3′E^* newborns.

### Cell type of the GFP-expressing kidney cells in *Epo^GFP/Δ3′E^* newborns

**Figure 3:**
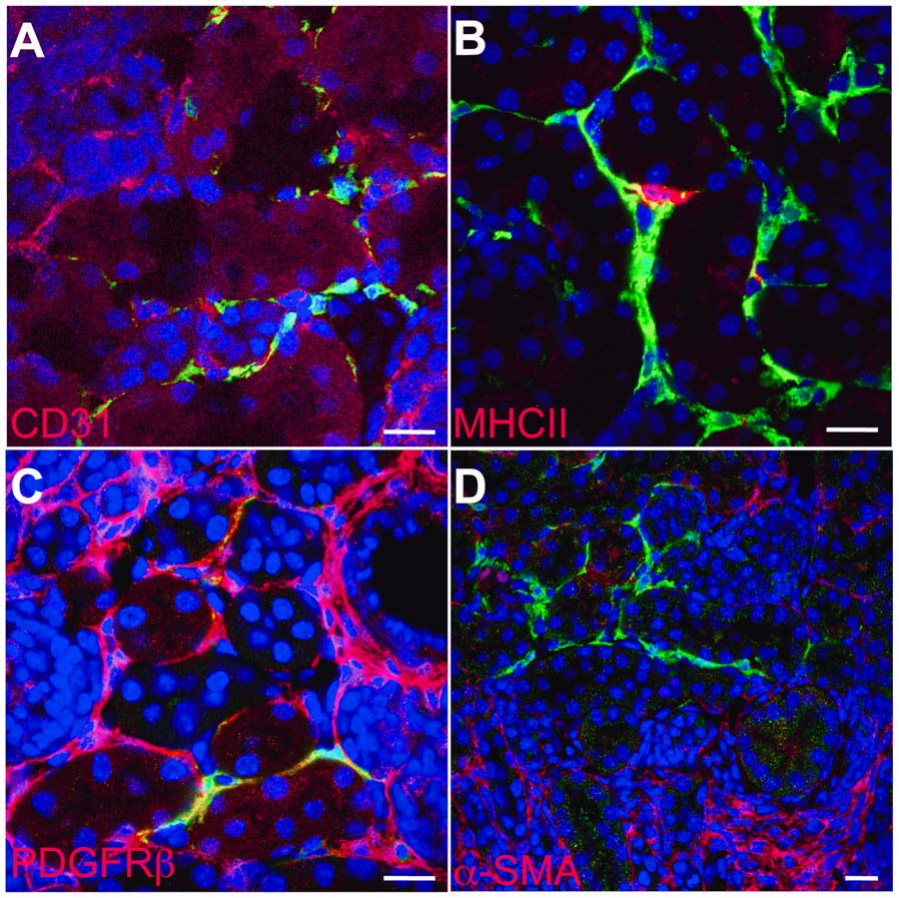
Cellular marker characterization of the GFP^+^ kidney cells of *Epo^GFP/Δ3′E^* mouse. Dual immunostaining combined with confocal microscopy indicating GFP-expressing cells are not endothelial (CD31, **A**), dendritic (MHCII, **B**), and tubular cells (E-cadherin, **Movie S1**). GFP-positive interstitial cells express fibroblast-like markers (PDGFRβ, **C**), but not myofibroblast marker (α-SMA, **D**). Kidney slices from P5 *Epo^GFP/Δ3′E^* mice were stained with GFP (green) and the indicated antibodies directly labeled with phycoerythrin (PE, red) or indirectly labeled with Alexa 594 (red) respectively. Merged images of the same kidney section are shown. DAPI: blue/nucleus; Scale bars: 25 µm.

**Figure 4:**
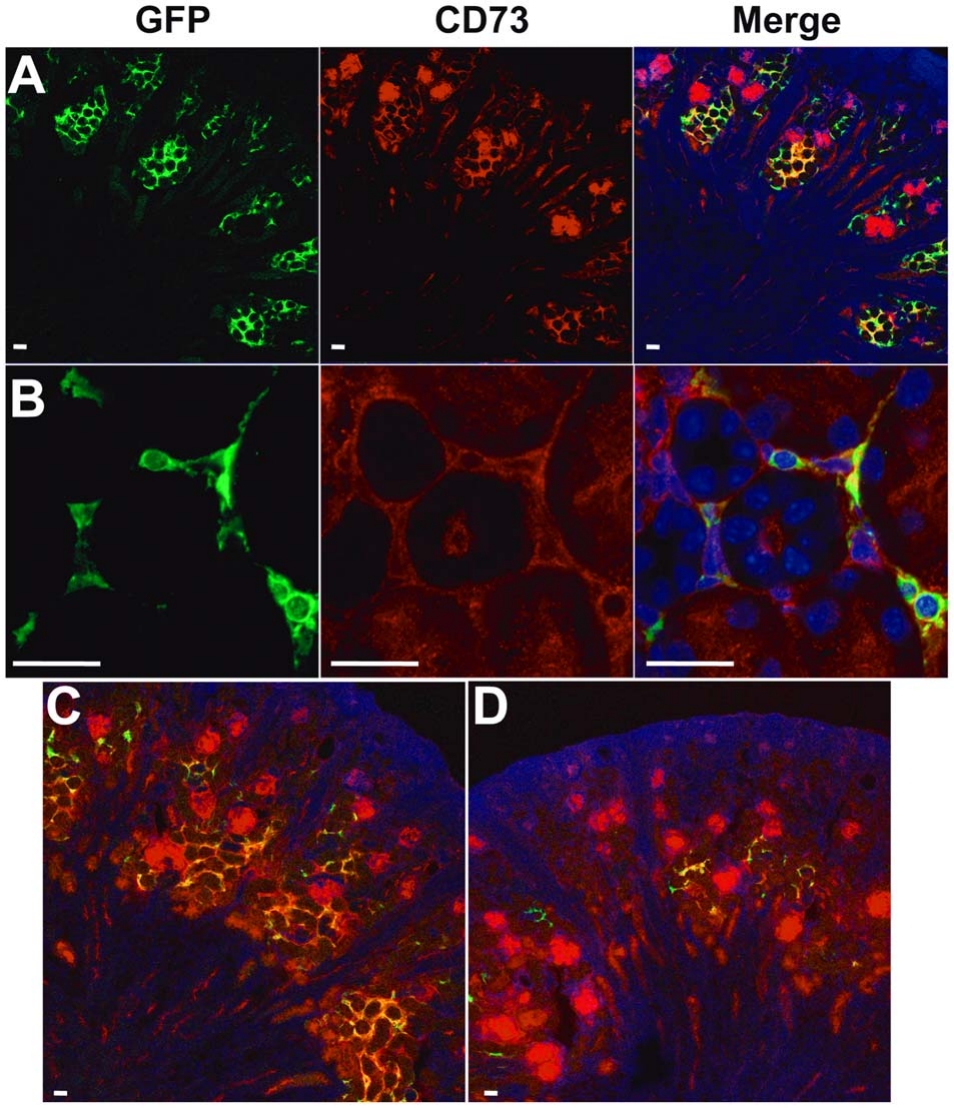
Co-localization of GFP and CD73 in the peritubular interstitial cells of the *Epo^GFP/Δ3′E^* kidneys. **A**–**C** Representitive images of confocal microscopy of kidney sections from P5 *Epo^GFP/Δ3′E^* mice. **B** A high-power view, demonstrated that GFP and CD73 were mostly co-localized in peritubular fibroblast cells (merged in yellow). Global merged view of the kidney sections from P5 *Epo^GFP/Δ3′E^* neonates (**C**), and *Epo^Δ3′E/wt^* littermates (**D**). **D** Under normal conditions CD73 showed a wide expression spectrum in a variety of cellular types including a few interstitial fibroblasts in the kidney. **C** During anemia note that CD73^+^ cells appeared to be increased mainly in the peritubular fibroblast population. GFP: green; CD73: red; DAPI: blue/nucleus; merged: yellow. Scale bars: 20 µm.

Kidney interstitium contains mainly fibroblastic, dendritic cells [28], and vascular endothelial as well as tubular cells have also been identified as the site of *Epo* gene expression in the kidney [5]. We therefore carried out marker studies on GFP-expressing kidney cells of P4–6 anemic newborns by dual immunostaining under confocal microscopy and FACS detection (Table 1). Expression of platelet-derived growth factor receptor β (PDGFRβ) (Figure 3C) and Ecto-5′-nucleotidase/CD73 (Figure 4), but not CD31 (Figure 3A), major histocompatibility complex class II (MHCII) (Figure 3B), or E-cadherin (Movie S1), indicated that REPs are cortical fibroblast-like, but not tubular, endothelial, or dendritic cells. Our three-dimensional (3D) movie reveals that REPs are tightly packed around tubules, but not tubular cells themselves (Movie S1). We also examined the expression of alpha-smooth muscle actin (α-SMA), a marker for the subtype of renal interstitial fibroblasts [28]. Staining with α-SMA antibody was observed in the medullar and cortical cells near the capsule, but did not overlap with GFP-positive cells (Figure 3D). Taken together, most neonatal REPs at P4–6 stage, are CD73-, PDGFRβ-positive, α-SMA-negative peritubular fibroblasts (Table 1). Thus, neonatal REPs have the typical characteristics of adult cortical fibroblasts previously reported in healthy rat kidney [29].

**Table 1:** Expression of cellular markers in REPs by multiple immunostaining methods.

Surface marker	Specificity in renal cells	Expression	Methods
E-cadherin	Tubule	-	CM*, FACS
CD31	Endothelium	-	CM, FACS
MHCII	Dendritic cell	-	CM, FACS
CD73	Interstitial fibroblast	+ (63%)	CM, FACS
α-SMA	Myofibroblast	-	CM
PDGFRβ (CD140b)	Interstitial fibroblast	+ (50%)	CM, FACS

*CM: Confocal Microscopy.

CD73 is considered to be a reliable marker for identification of renal cortical fibroblasts [27], and *Epo*-expressing cells [6], [11], [12], [28], [30]. In the kidney sections of P4–6 *Epo^GFP/Δ3′E^* newborns, roughly 60%, but not all of GFP-positive cells, expressed CD73. Therefore, GFP-positive cells were divided into CD73^+^ and CD73^−^ populations (Figure 4A–C). Both CD73^+^ and CD73^−^ GFP-expressing cortical interstitial cells were remarkably increased in *Epo^GFP/Δ3′E^* neonates (Figure 4C) compared with in *Epo^Δ3′E/wt^* littermates (Figure 4D). In *Epo^Δ3′E/wt^* non-anemic kidney, only a few GFP-expressing cells constituting both CD73^+^ and CD73^−^ were observed and located in the juxtamedullary layer of the cortex (Figure 4D).

Unlike GFP signals, which were restricted to peritubular interstitium, CD73 stains were widely spread in the renal cortex. Besides fibroblasts, proximal tubule (brush boarder), glomeruli (mesangial cells) and the cells in the medullar rays were also observed to express CD73 in sections. The proportion of CD73 expressing cells and their staining intensity in non-fibroblast cells were similar in both anemic and non-anemic kidney sections (Figure 4C, D), *i.e.* among various types of CD73-expressing kidney cells, only cortical fibroblasts showed a hypoxia/anemia-responsive tendency.

### FACS sorting of GFP^+^ cells and *Epo* mRNA expression in the isolated cells

**Figure 5:**
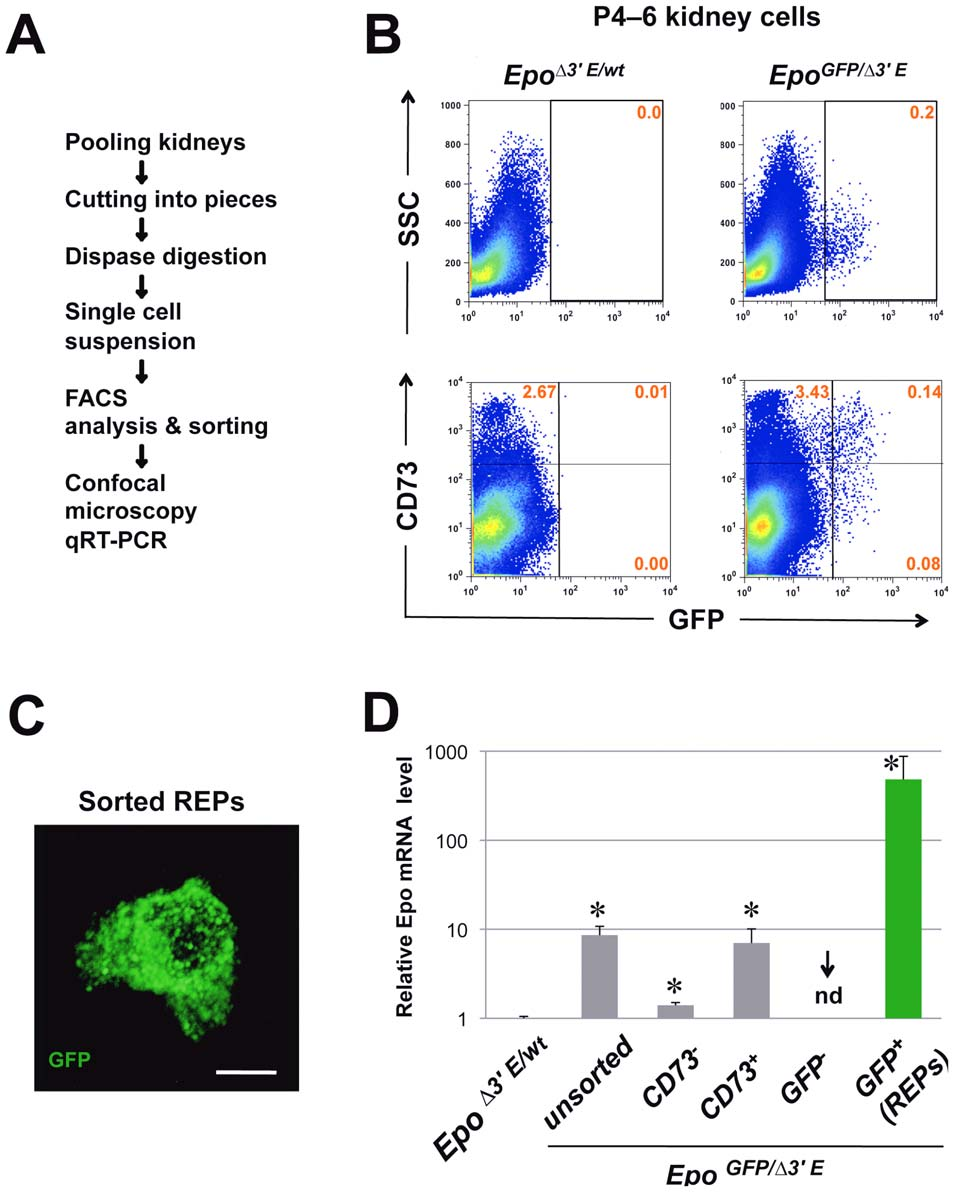
Isolation of REPs from the P4–6 *Epo^GFP/Δ3′E^* anemic kidneys. **A** Cell purification protocol for a rare population of REPs from kidney. **B**, **upper panel** Representative FACS scatter plot of kidney cells from pooled P4–6 *Epo^GFP/Δ3′E^* neonates demonstrating GFP^+^ cells above the gate (set using *Epo^Δ3′E/wt^* control cells). **B**, **lower panel** Assessment by FACS of percentage of GFP^+^ and CD73^+^ fractions in the kidney cells from P4–6 anemic *Epo^GFP/Δ3′E^* neonates. Pooled results were from three independent experiments. Note CD73 expression divides GFP^+^ cells into two parts: CD73^+^ and CD73^−^. **C** Representative confocal microscopy of the FACS-sorted GFP^+^ cells (REPs) with anti-GFP immunostaining. Scale bar: 5 µm. **D** Analysis of relative *Epo* mRNA levels by qRT-PCR (*Hprt* as a loading control) in FACS-purified GFP^+^ or the remaining GFP^−^ kidney cells from P4–6 *Epo^GFP/Δ3′E^* mice; CD73^+^ vs. CD73^−^ fractionated cells were also evaluated. The data shown are from four experiments, each performed in duplicate. nd: not detectable; *p<0.05.

We isolated GFP^+^ cells from the kidneys of P4–6 *Epo^GFP/Δ3′E^* neonates by flow cytometry. Kidney cells from *Epo^Δ3′E/wt^* littermates were used as negative controls (Figure 5B, upper-left panel). The GFP^+^ population was present grossly with a low to intermediate GFP intensity and constituted up to 0.2% of the total fresh kidney cells (Figure 5B, upper-right panel). This yielded several thousands viable GFP^+^ cells per *Epo^GFP/Δ3′E^* mouse at P4–6 newborn stage, with a purity of greater than 75%. We also assessed the association of GFP-expressing cells with CD73. As shown in Figure 5B, lower-right panel, of GFP^+^ cells from P4–6 anemic kidneys, 63% were CD73^+^ and 37% were CD73^−^. A confocal microscopic image of the sorted GFP^+^ cells is shown with anti-GFP immunostaining (Figure 5C).

Subsequently, we evaluated the expression of *Epo* mRNA in each sorted fraction from P4–6 *Epo^GFP/Δ3′E^* kidneys, and in unsorted kidney cells of P4–6 *Epo^Δ3′E/wt^* and *Epo^GFP/Δ3′E^* newborns as well. The hypoxanthine-phosphoribosyl-transferase (*Hprt*) gene was used as a loading control, because expression of HPRT is less affected by hypoxia/anemia [31]. As shown in Figure 5D, qRT–PCR revealed high *Epo* mRNA expression exclusively in samples from *Epo^GFP/Δ3′E^* animals including unsorted kidney cells, CD73^−^, CD73^+^ and GFP^+^ subsets. In *Epo^GFP/Δ3′E^* mice, compared with the unsorted kidney, *Epo* mRNA levels were ∼100 fold enriched in the GFP^+^ fraction, but not in the CD73^+^ fraction. *Epo* mRNA expression was low but detectable in *Epo^Δ3′E/wt^* kidney cells at P4–6, but not in the GFP-negative fraction of *Epo^GFP/Δ3′E^* kidney cells (Figure 5D). We have reported that neuronal markers are expressed by Epo-producing cells in the adult kidney [6]. Consistently, transcripts for microtubule-associated protein 2 (MAP2) and neurofilament light polypeptide (NFL) were detected in the GFP^+^ fraction from P4–6 *Epo^GFP/Δ3′E^* kidneys (data not shown). These results demonstrate our system for isolation of REPs is reliable and efficient.

### Gene expression profile of oxygen-sensing and HIFs of the isolated REPs

**Figure 6:**
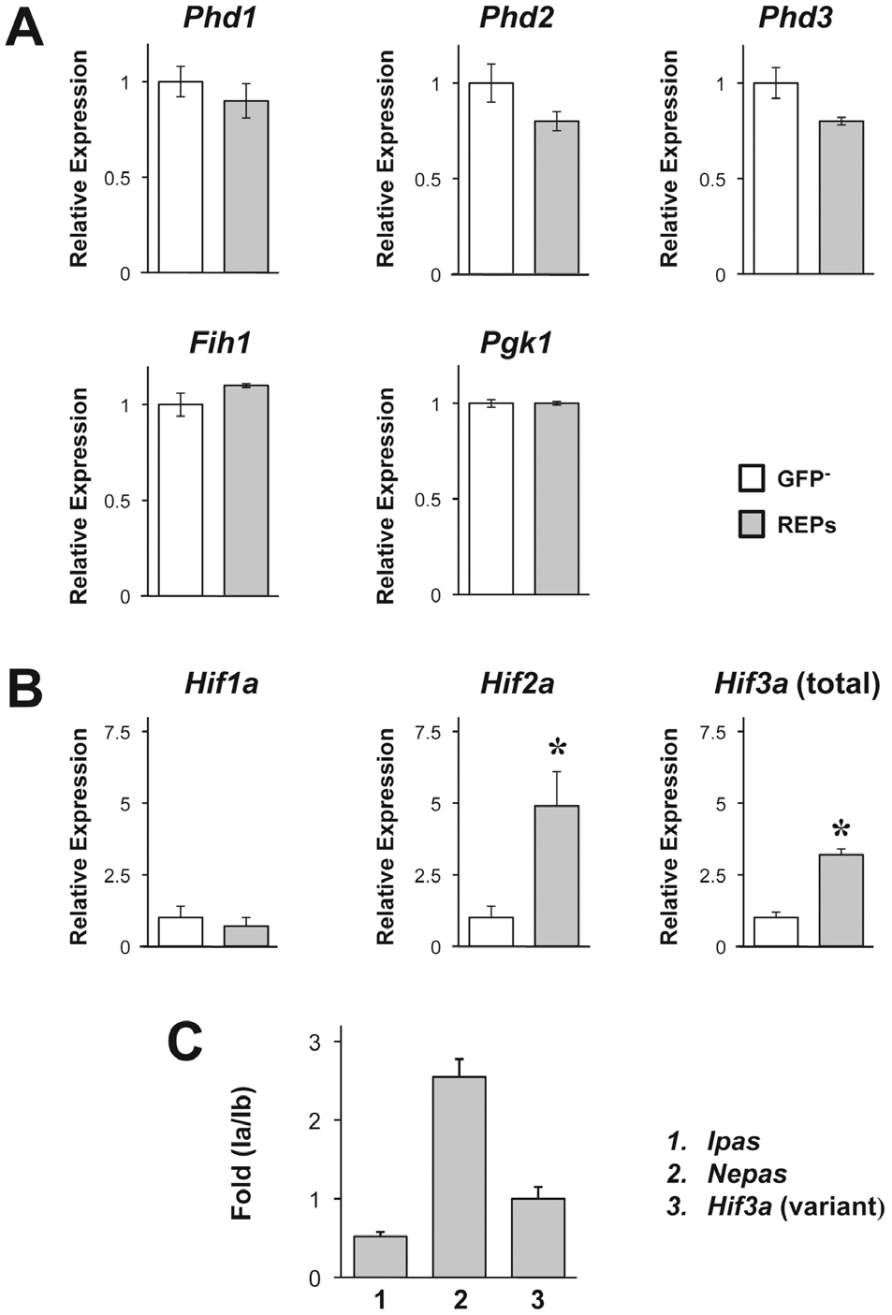
Expression profile of cellular oxygen-sensing and hypoxia-inducible molecules in the isolated REPs. qRT-PCR analysis of the genes related to hypoxia response using RNA extracted from the sorted GFP^−^ and GFP^+^ renal cells from P4–6 *Epo^GFP/Δ3′E^* mice. *Hprt* as a loading control *p<0.05. Data are for three experiments performed in duplicate. **A** Four genes related to cellular oxygen-sensing; **B** three *Hifα* isoforms. Also note that HIF1α targets: *Pgk1*, *Phd2* are not enriched in the GFP^+^ fraction (REPs). **C** Three transcript variants of *Hif3α* in the purified GFP^+^ fraction (REPs), using a method for exponential PCR amplification at a fixed threshold (see also **Materials and Methods**).

We examined the expression of molecules, known to be involved in oxygen tension-dependent regulation [19], by qRT-PCR analysis. Compared to GFP-negative kidney cells, no enrichment of mRNA expression of oxygen sensor genes, *Phd1*–*3* and *Fih1* genes was found in the REPs fraction (Figure 6A). *Hif2α* but not *Hif1α* (Figure 6B, upper panel) mRNA expression was up-regulated in the REPs. In this line, no enrichment of HIF1α target genes [4], *Pgk1* and *Phd2*, *Phd3* were found in the REPs. These are consisted with recent reports on the relationship of HIF2α to renal Epo production [12], [29], [32], [33].

*Hif3α* mRNA expression was also enriched in the REPs ((Figure 6B, upper right panel). In the three alternatively spliced variants of *Hif3α* mRNA: *Ipas*, *Nepas* and *Hif3α*, *Nepas* is expressed during infantile and newborn stages, while *Ipas* is seen in adults. In accordance with a reported method [34], [35], we examined the expression of these splice variants in REPs, and detected all three variant mRNAs in the fraction of REPs at P4–6 stage. *Nepas* mRNA expression was the highest, suggesting *Nepas* might be a dominant form among the three splicing variants of the *Hif3α* at P4–6 infantile stage (Figure 6C).

## Discussion

By generating *Epo^GFP/Δ3′E^* mice, we isolated a specific type of renal cells namely REPs, which are responsible for Epo production after birth. REPs 1) are fibroblast-like interstitial cells residing in the tubulo-interstitial compartment of the kidney in anemic hosts (Hct 18%); 2) constitute up to 0.2% of whole kidney cells. About 63% of REPs also express CD73, a marker for cortical fibroblasts and *Epo*-expressing cells in kidney [6], [10], [28], [30]; 3) highly express *Hif2α*, *Hif3α*, but not *Hif1α* mRNAs; 4) are efficiently isolated from naive kidney tissues as GFP-expressing cells in our mutant.

### Isolation system of REPs

Previously, difficulties in the isolation of REPs prevented better understanding of the mechanisms of *Epo* regulation in response to hypoxia. We developed an isolation system that phenotypically labeled REPs in the kidney, by way of a *GFP* knock-in (*Epo^GFP^*) combined with a 3′ enhancer (*Epo^Δ3′E^*). This enabled us to purify REPs, a rare cell population, from kidney by FACS-sorting.

Our GFP knock-in strategy facilitates the capacity to express GFP based on endogeneous *Epo* gene expression confined to a rare population, without worries about aberrant or ectopic transgene expression. As a result, we identified REPs as peritubular fibroblast-like interstitial cells concentrated at the cortico-medullary junction, corresponding to our previous finding from the *Epo-GFP* transgenic mouse studies [5], [6].

In the history of Epo research, *in situ* hybridization is a classic method to localize *Epo* mRNA; but extensive studies lead to confusion of REPs' whereabouts: peritubular interstitium, or a tubular site [5]. Transgenic mice, created by integrating a marker gene with regulatory sequences of the *Epo* gene, have provided a powerful tool for accurate localization of REPs in the kidney [6], [8], [11]. In mice bearing an *Epo*/*SV40 T* antigen transgene, REPs were successfully identified but attempts to isolate REPs *in vitro* failed [8], [11]. Importantly, later transgenic mouse studies suggested that a much wider (20-kb∼) flanking region of the *Epo* gene was needed for adequate levels of transgene expression in kidney [5], [6], [36].

To induce *Epo* gene expression, pre-treatment to induce hypoxia/anemia is usually required. These procedures, such as bleeding or phenylhydrazine injection, are not always successful in inducing stable anemia. For instance, we have tried isolation of REPs using our *Epo*-*GFP* transgenic mouse [6]. The GFP^+^ population of the kidney cells from the transgenic mouse were not distinct under FACS detection, despite that severe anemia was induced by phlebotomy. *Epo^GFP/Δ3′E^* mice provide a handy and important source: lacking the 3′ enhancer demonstrated impaired hepatic *Epo* expression and profound anemia (Hct value was about 18% in newborn stage P4–6) and allowed us to directly sort REPs by FACS, which are constantly and stably labeled with GFP fluorescence in newborn kidneys. Moreover, considering the loose connections of renal tissues and the decreased interstitial volume of the cortex, renal tissues from newborns promised to be a better source of this rare cell population than adult kidney [37]. What is important that newborn REPs are fully functional with regard to Epo secretion, soon after birth [9], [23]. Indeed, newborn REPs (P4–6) displayed adult REPs phenotypes: fibroblast-like (CD73^+^/α-SMA^−^) [28], [29] with neuronal marker expression (MAP2^+^/NFL^+^) [6].

### Cellular characters of the GFP-labeled REPs

GFP coupled with marker molecule analyses revealed that REPs are localized in the deep renal cortex, form a network around tubules and adjacent capillaries with their processes, and express fibroblast markers CD73, PDGFRβ and soluble guanylyl cyclase (sGC) (data not shown) but not other cell markers CD31, MHCII or E-cadherin. Surface expression of these molecules on REPs was verified by both confocal microscopy and flow cytometry. Therefore, all of these data support a widely accepted notion that REPs are peritubular fibroblast-like interstitial cells [6], [10], and resolves earlier conflicts on the cell identity in the literature [5].

Fibroblasts are considered to be an easy cell type to cultivate. However our attempts to culture REPs were not successful. It is interesting to consider that REPs are a type of fibroblasts in a resting state, and when induced to proliferate (signified by α-SMA expression), they lose their ability to produce Epo [38], [39], such as the case for renal fibrosis and renal anemia. Concurring with our previous finding that adult REPs express neuronal markers [6], we confirmed *Map2* and *Nfl* mRNA expression in FACS-sorted REPs from P4–6 newborns. These observations further support the notion that REPs are unique fibroblast-like cells.

### Association with CD73 during anemia

Our histological and flow-cytometrical examinations revealed that REPs constitute grossly 0.2% of total kidney cells, with 63% co-expressing CD73 in the case of our *Epo^GFP/Δ3′E^* mice (Hct 18% at P4–6 newborn stage). In the non-anemic kidneys, only a few REPs, composed of both CD73^+^ and CD73^−^, can be observed in the juxtamedullary layer of the cortex. These cells probably represent a basal level of Epo production under normal conditions, which is required for daily production of red blood cells in normal individuals. In anemic kidneys both CD73^+^ and CD73^−^ REPs are robustly increased in a pattern that spreads outward from the deep cortex toward the capsule and the inner medullar.

qRT-PCR revealed a 100-fold enrichment of *Epo* mRNA in the GFP^+^ fraction, but no enrichment in the CD73^+^ fraction, compared with the unsorted total kidney cells. CD73 has a wide expression profile in renal cortex, occupying roughly 3% of the total kidney cells at P4–6 newborn stage based on our FACS study. In addition to the interstitial cells, such as fibroblasts, T-, B-lymphocytes, many parenchymal cells *e.g.* glomorular mesangial, proximate tubular (brush board), collecting duct cells etc. also express CD73 [28]. Comparing the pattern of CD73 staining in the anemic kidney section with the non-anemic one, it seems that anemia increases the number of CD73^+^ cortical interstitial fibroblasts but not other types of CD73^+^ cells. Because cortical fibroblasts are rare population, among all of the CD73^+^ cells, the percentage of CD73 fraction did not change much in anemic kidneys compared with non-anemic kidneys. The heterogeneity of CD73 expression in REPs may reflect different functional or matured stages during anemia [39]]. Recently, it has been reported that loss-of-CD73 does not affect the expression of Epo [40]. Our data that only a part of REPs express CD73 in anemic kidneys seem to conform this finding that the loss of CD73 has not impact on renal erythropoietin induction under hypoxia.

### Expression profile of oxygen sensor molecules and transcriptional determinants in REPs

Clinically, congenital defects of the oxygen-sensing pathway have been reported including *VHL*, *PHD2* and *HIF2A* mutations that cause secondary erythrocytosis through the *EPO* gene over-expression [1], [7]. PHD2 inactivation is sufficient to induce near max. renal Epo production [41], [42]; and recent RNAi-based studies confirmed the major role of PHD2 in Epo regulation *in vitro* as well as *in vivo* [43]. Human and rat PHD2 mRNA are hypoxically induced by HIFs for negative feedback regulation [44], [45]. In the carbon monoxide exposed rat, PHD3 protein was detected and co-localized with HIF2α in cortical interstitial cells of the kidneys [46]. *Phd2*, *3* are the targets of HIF1α [4]. In this study, we examined four genes encoding oxygen-sensor molecules (PHD1–3 and FIH1) and did not observe any enrichment in REPs compared with other cells of the anemic kidneys in our gene expression profiling. This may be because all of these four genes are ubiquitously expressed in kidney, with respect to various cell types of the kidney.

As described above, *Hif2α*, rather than *Hif1α* shows highly REPs-specific expression patterns at the mRNA level. Interestingly, *Hif2α* mRNA levels are particularly high in tissues that are important for the systemic delivery of oxygen, for example the lung, heart, endothelium and the carotid body [47]–[49]. Quite recently, HIF2α protein expression has been shown in the *peritubular fibroblasts* that express *Epo* and CD73 in rat kidneys [30]. Preferential binding of HIF2α protein to the HRE within the native *Epo* gene 3′ enhancer has been also confirmed in hepatocytes [50]. As renal *Epo* expression does not depend on the *Epo* gene 3′ enhancer [23], the existence of a possible renal enhancer with a different HRE awaits investigation.

Enrichment of *Hif3a* mRNA was also observed in the REPs. Transcripts of all of the three splicing variants (*Nepas*, *Ipas* and *Hif3a*) of *Hif3a* could be detected in newborn REPs (P4–6), where *Nepas* seems to be the dominant form of the three. *Nepas* and *Ipas* have been demonstrated to be hypoxia-induced factors due to the presence of functional HREs upstream of Exon1a, and act as negative regulators of the HIF pathway. Both *Ipas* and *Nepas* show a cell-, and stage-specific expression pattern [21], [22]. IPAS (inhibitory PAS protein) has already been reported to work as a negative feedback factor in a hypoxic condition in the cornea [21], [51], but there is no literature on its roles in hematopoiesis so far. Our targeted *Hif3a* knockout mice (*Hif3a^−/−^*) show an impaired cardiovascular formation around birth. This phenotype is possibly caused by over-expressed *Endothelin-1* in pulmonary endothelium. HIF3α (Nepas) was suggested to suppress HIF2α-driven transcription of *Endothelin-1* according to the localization and reporter assays [22].

We were curious to see if a similar mechanism exists in *Epo* gene regulation. We are starting to explore the function of HIF3α in erythropoiesis by examination of our established *Hif3a^−/−^* mice [22]. *Hif3a^−/−^* mice were viable and fertile without abnormalities under normal conditions. Based upon our preliminary data in mouse hypoxia experiments, it appeared that *Epo* transcript showed up-regulated tendency in *Hif3a^−/−^* kidneys, in contrast to the wild type counterparts. In a recent review, McIntosh *et al.* also mentioned an erythropoietic phenotype in their independent *Hif3a^−/−^* mice [52]. We, therefore, hypothesized HIF3α-related negative regulation is also necessary in renal Epo production during hypoxia/anemia. By this, homeostasis of red blood cell mass might be maintained to prevent erythrocytosis and thrombosis occurring in animals and human beings. HIF response to hypoxia is complex. A recent report has demonstrated that human *HIF3A* gene expression is induced by hypoxia through activation of HIF1α but not HIF2α [53]. It raises the possibility that in REPs, *Hif3a* mRNA expression might be up-regulated by HIF2α, because REPs preferentially express *Hif2a* rather than *Hif1a*.

Recently a renal cell line producing Epo with a hypoxia-dependent manner has been successfully established from a patient suffering from renal cancer [12]. Here, we report for the first time on isolation or purification of REPs *in vivo*. Our mouse enables the purification of a rare cell population specific for renal *Epo* expression during anemia and a detailed examination of the hypoxia-dependent aspect of the cells. Finally, we report the novel finding that *Hif2α* and *Hif3α* (but not *Hif1α*) mRNA are preferentially expressed in REPs. Combined with recent evidence *in vivo* about the role of HIF2α in erythropoiesis, we propose a hypothesis: positive regulation by HIF2α and negative regulation by HIF3α may be necessary for correct renal *Epo* induction during hypoxia/anemia.

## Materials and Methods

### Generation of *Epo^GFP/Δ3′E^* mice

All mice used were from a C57BL/6 genetic background and were strictly kept in the specific-pathogen-free conditions. All experiments were conducted in accordance with the regulations of The Standards for Human Care and Use of Laboratory Animals of Tohoku University. The protocol was approved by the Committee on the Ethics of Animal Experiments of Tohoku University (Permit Number: 21-Idou-144 and 22-Idou-113).

*Epo^GFP/Δ3′E^* mice were generated by mating mice heterozygous for *Epo^GFP/wt^* with mice homozygous for deletion of the 3′enhancer (*Epo^Δ3′E/Δ3′E^*) [23]. Genotyping was performed by polymerase chain reaction (PCR) with the primer sets listed in Table 2. From this mating, half of the offspring would be *Epo^GFP/Δ3′E^* mice. These mice are genetically deficient in *Epo* gene 3′enhancer activity and had anemia within two weeks after birth.

**Table 2:** Oligo-nucleotide sequences of primers used in this study.

Use	Sequence (5′-3′)
Genotyping of the *Epo^GFP^* allele	ACTCTCGGCATGGACGAGCTG
	GTGAGTGTTCGGAGTGGAGCAGG
Genotyping of the *Epo^Δ3′E^* allele	ACATGGTCCTGCTGGAGTTC
	ACACACTCCCAGCAAATTCC
Genotyping of the *Epo* gene (3′ enhancer)	CAGGCTCCATTCAAGGC
	CCTGCAGTGGACTTTGAAGGC
Genotyping of the endogenous *Epo* gene	GGCATGGCTCAATGATTAGG
	GTGAGTGTTCGGAGTGGAGCAGG
qRT-PCR for *Hif1α* mRNA	CCTGCACTGAATCAAGAGGTTGC
	CCATCAGAAGGACTTGCTGGCT
qRT-PCR for *Hif2α* mRNA	GGACAGCAAGACTTTCCTGAGC
	GGTAGAACTCATAGGCAGAGCG
qRT-PCR for *Hif3α* mRNA (total)	AAGACGCCCTGACCCCCAGG
	CCCTCTGCTGGTGAGCGTGC
qRT-PCR for *Ipas* mRNA	TCCACGATGGTGCTACTCTG
	TGTCCTTCACTCCCTCCTAG
qRT-PCR for *Nepas* mRNA	CTGCAGCGCGTGAGGTCG
	CTTTTTCCACCTGGTTCCAC
qRT-PCR for *Hif3α* mRNA (variant 1)	ACCAAGACAGGTCGAACACC
	TTTTCCACCTGGTTCCACTC
qRT-PCR for *Phd1* mRNA	ATGGCTCACGTGGACGCAGTAA
	CATTGCCTGGATAACACGCCAC
qRT-PCR for *Phd2* mRNA	TAAACGGCCGAACGAAAGC
	GGGTTATCAACGTGACGGACA
qRT-PCR for *Phd3* mRNA	CTATGTCAAGGAGCGGTCCAA
	GTCCACATGGCGAACATAACC
qRT-PCR for *Fih1* mRNA	CGAAGTTACAGCTTTCCGACCAG
	GTTTGTGTCGGTCAGCACCACT
qRT-PCR for *Pgk1* mRNA	GATGCTTTCCGAGCCTCACTGT
	ACCAGCCTTCTGTGGCAGATTC
qRT-PCR for *Epo* mRNA	CATCTGCGACAGTCGAGTTCTG
	CACAACCCATCGTGACATTTTC
qRT-PCR for *Hprt* mRNA	GTTGGATACAGGCCAGACTTTGT
	CCACAGGACTAGAACACCTGC

### Hematological analysis

Whole blood was collected from the carotid arteries, and hematopoietic indices were measured using an automatic blood cell analyzer (Nihon Koden).

### Immunostaining

Kidneys were immersion-fixed in 4% paraformaldehyde (Nakarai Tesque) for 3 hours at 4°C and embedded in OCT compound (Sakura Finetechnical). Frozen sections 20 µm in thickness were incubated with primary antibody for 16 hours at 4°C, and detected by Alexa Fluor 488 (Molecular Probes) or Alexa Fluor 555 (Molecular Probes) conjugated anti-IgG as second antibodies. Color detection was performed using diaminobenzidine as a chromogen (brown color staining). Nuclei were stained with 4′-diamidino-2-phenylindole (DAPI). Fluorescent images were observed using the LSM510 confocal imaging system (Carl Zeiss).

All of the primary antibodies were diluted 1∶500 in blocking solution (Dako) as follows: Anti-GFP (MBL); Biotinylated Anti-E-Cadherin (R&D systems); anti-CD31 (BD Pharmingen); phycoerythrin (PE) anti-CD73 antibody (BD Pharmingen); Alexa Fluor 647 Anti-CD73 (Biolegend); anti-α-SMA (Abcam); Allophycocyanin (APC) anti-CD140b (PDGFRβ) (Biolegend); APC anti-MHCII (eBioscience).

### FACS analysis and cell sorting

*Epo^GFP/Δ3′E^* anemic newborns were sacrificed at P4–6. The kidneys were collected in PBS from several litters and teased away from their surrounding tissues. Single cell suspension was prepared using dispase (1.25 mg/mL; Invitrogen) in PBS–15% FCS for 60 min at 37°C followed by washing in DMEM-Ham's F-12–10% FCS and passing through a nylon mesh to remove any clumps. This cell preparation was approximately 85% single viable cells. Whole kidney was analyzed and sorted on the flow cytometer (FACSAria, Becton Dickinson). The effectiveness of each FACS separation was assessed by immediately resorting an aliquot of GFP^+^ and GFP^−^ cells (data not shown). Greater than 75% of the GFP^+^ population resorted to the same gate used in the initial sort.

### qRT-PCR analysis

Total RNA was extracted from FACS-purified cells using Isogen reagent (Nippon Gene), according to the manufacturer's protocol. RNA was then concentrated using RNeasy MinElute columns (Qiagen) and first strand cDNA synthesis was performed using the SuperScript III First Strand Synthesis System for RT-PCR (Invitrogen).

Primers for amplifying 100–300 bp of each PCR product were used (Table 2). PCR reactions were SYBR Green programmed and carried out using qRT-PCR Mastermix (Takara). Each sample was analyzed in duplicate or triplicate. The data were normalized by subtracting the difference of the C_T_ values between the target genes of interest (Tgene) and that of *Hprt* mRNA, thereby obtaining a ΔC_T_ (Tgene C_T_−HPRT C_T_). Relative expression (fold induction) was calculated as 2^−(SΔC^_T_^−CΔC^_T_^)^ where SΔC_T_−CΔC_T_ is the difference between the sample ΔC_T_ (GFP^+^ cells) and the control ΔC_T_ (GFP^−^ cells). Both target gene and *Hprt* reactions approached 100% efficiency as determined by standard curves. PCR products were analyzed by dissociation curve and on agarose gels to check that a single band was amplified.

The molar ratio was calculated as previously described [33], [34]: molar ratio = [*L*_a_×(1+*E*_a_)^CTa^]/[*L*_b_×(1+*E*_b_)^CTb^]. *L*_a_ and *L*_b_ indicate lengths of the amplicon for 1a and 1b transcripts, respectively. *E*_a_ and *E*_b_ indicate the amplification efficiency of a primer set for *1a* and *1b* transcripts, respectively. CT_a_ and CT_b_ indicate the numbers of threshold cycles for the *1a* and *1b* transcripts, respectively.

### Statistics

Statistical analysis was performed between samples and controls using *t*-test (two tailed, unequal variance, *p*≤0.05 cut-off).

### Supporting Information

3D-movie of REPs and their spatial coordination, were made by compiling images collected using a Zeiss LSM 510 confocal microscope. One *z*-slice of the stack is shown in Movie S1.

## Supporting Information

Movie S1 **3D image of REPs by confocal laser-scanning microscopy.** Kidney sections from P5 *Epo^GFP/Δ3′E^* newborn were co-stained with anti-E-cadherin to label the tubular cells. GFP: green; E-cadherin: white; DAPI: blue/nucleus. Scale bar: 20 µm.
(MOV)
